# Endoscopic injection of bulking agents in pediatric vesicoureteral reflux: a narrative review of the literature

**DOI:** 10.1007/s00383-023-05426-w

**Published:** 2023-02-18

**Authors:** Maria Escolino, Nicolas Kalfa, Marco Castagnetti, Paolo Caione, Giovanni Esposito, Luisa Florio, Ciro Esposito

**Affiliations:** 1 Pediatric Surgery Unit, Federico II University Hospital, Via Pansini 5, 80131 Naples, Italy; 2 Pediatric Surgery Unit, University Hospital of Montpellier, Montpellier, France; 3 Pediatric Urology Unit, Bambino Gesù Children Hospital, Rome, Italy; 4 Pediatric Urology Unit, Salvator Mundi International Hospital, Rome, Italy; 5 CEINGE, Advanced Biotechnologies, Naples, Italy

**Keywords:** Vesicoureteral reflux, Endoscopy, Injection, Bulking agent, Technique, Children

## Abstract

In the last 20 years, endoscopic injection (EI) has affirmed as a valid alternative to open surgery for management of pediatric vesicoureteral reflux (VUR). This study aimed to investigate and discuss some debated aspects such as indications, bulking agents and comparison, techniques of injection and comparison, predictive factors of success, use in specific situations. EI is minimally invasive, well accepted by patients and families, with short learning curve and low-morbidity profile. It provides reflux resolution rates approaching those of open reimplantation, ranging from 69 to 100%. Obviously, the success rate may be influenced by several factors. Recently, it is adopted as first-line therapy also in high grade reflux or complex anatomy such as duplex, bladder diverticula, ectopic ureters. The two most used materials for injection are Deflux and Vantris. The first is absorbable, easier to inject, has lower risk of obstruction, but can lose efficacy over time. The second is non-absorbable, more difficult to inject, has higher risk of obstruction, but it is potentially more durable. The two main techniques are STING and HIT. To date, the ideal material and technique of injection has not yet clearly established, but the choice remains dependent on surgeon’s preference and experience.

## Introduction

Endoscopic injection (EI) has reported widespread use in the last two decades for treatment of pediatric vesicoureteral reflux (VUR), becoming a valid alternative to open surgery and continuous antibiotic prophylaxis (CAP). The main reasons are that this option treatment is minimally invasive, can be performed on an outpatient basis, and has a relatively short learning curve and low complication rate [1].

Considerable advancements have been made regarding the materials used and the injection techniques. To date, dextranomer/hyaluronic acid (Dx/HA, Deflux, Salix Pharmaceuticals, NJ, USA) is the most widely adopted bulking agent approved by the US Food and Drug Administration (FDA), with an overall mean success rate of 83% [1].

However, controversies on the use of EI have emerged with respect to the reporting of long-term success rates and delayed complications. Furthermore, little evidence is currently available regarding the efficacy of EI in preventing urinary tract infections (UTIs) and VUR-related renal damage.

This study aimed to review the current state of the art of EI treatment and provide an updated overview of this topic. More specifically, our purpose was to investigate and discuss the following points: (1) indications; (2) bulking agents and comparison; (3) techniques of injection and comparison; (4) predictive factors of success; (5) specific situations; (6) controversies.

## Materials and methods

An electronic literature search of PubMed was performed for the present study. Search terms utilized were as follows: “endoscopic injection” AND “vesicoureteral reflux” AND “bulking agent” AND “technique” AND “pediatrics”. The inclusion criteria were: all types of articles, articles published in PubMed, and related only to pediatrics. The exclusion criteria were: articles for which full text was not available, those with content redundancy and not written in English. From the articles retrieved in the first round of search, additional references were identified by a manual search among the cited references.

## Results

**Fig. 1:**
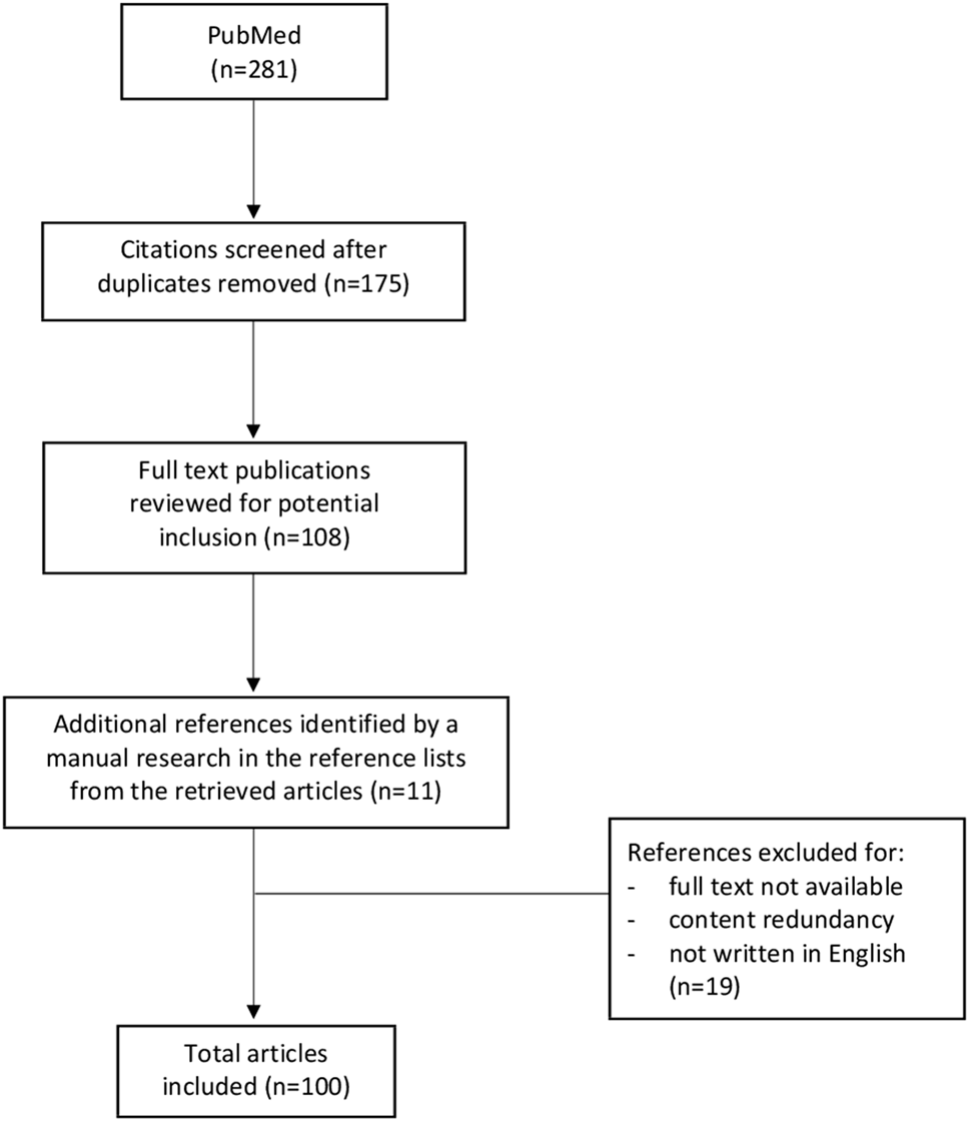
Flowchart of the literature selection process for the present article

One hundred articles, published over the period 1981–2022 and reporting on endoscopic injection treatment of pediatric VUR, were obtained. Figure 1 reports the flowchart of the literature selection process for the present article.

### Evidence-based medicine (EBM) and VUR treatment

Current gold standard surgical option for pediatric VUR is open trans-hiatal ureteral reimplantation according to Cohen with very high success rate (98%) [2]. Endoscopic injection (EI), laparoscopic extravesical ureteral reimplantation (LEVUR) and robot-assisted laparoscopic ureteral reimplantation (RALUR) are alternatives to open approach [3, 4]. One randomized controlled trial (RCT 2b-C) [5] comparing Cohen’s reimplantation vs EI in children aged over 1 year reported short- and long-term outcomes similar for VUR grades II, III, and IV. However, limitations of the study included limited size of the sample and multiples EI in 28% of cases.

Three long-term studies (low LE) were published [6–8], with a follow-up period ranging from 3 to 22 years.

Different studies [9–14] analyzed the main predictors for EI failure: VUR grade, injection technique, surgeon experience, patient age, renal scar at time of treatment, presence of untreated bowel bladder dysfunction (BBD), radiologic features and anatomical factors (distal ureteral diameter ratio).

### Natural history of VUR and indications to endoscopic injection

Most VUR may not be operated on. There is the chance of spontaneous resolution for pediatric VUR and the possibility to predict the resolution rate using specific scoring tools. Kirsch et al. [15] designed VUR index (VURx), a simple scoring tool to identify factors associated with VUR resolution in children less than 2 years of age and predict improvement and resolution. Children older than 2 years, with grade 4–5 reflux, complete ureteral duplication or periureteral diverticula, and VUR on filling phase, as well as female gender, had significantly (*p* < 0.01) longer time to improvement or resolution on multivariate survival analysis. VURx 1 to 5–6 had improvement/resolution rates of 89, 69, 53, 16% and 11%, respectively. Female gender, high-grade VUR, ureteral anomalies, and filling reflux are associated with longer time to improvement and non-resolution. Sjöström et al. [16] provided a scoring system for predicting downgrading and resolution of infantile high-grade VUR (> grade 3). A scoring system with a total of 14 points was built from four independent risk factors (gender, breakthrough UTI, type of renal damage and subnormal glomerular filtration rate). Children with persistent VUR (grade 3–5) had higher scores compared with the group with spontaneous resolution (grade 0–2) (*p* < 0.0001). A score of ≥ 8 points indicated a low probability of VUR resolution (≤ 14%) and confirmed indication to EI treatment.

Another important factor potentially influencing the outcome of endoscopic injection is represented by concomitant VUR nephropathy. Approximately 10–15% of patients prenatally diagnosed with reflux have renal scars and 30% of patients prenatally diagnosed with reflux have bowel bladder dysfunction (BBD) and renal scars [17, 18]. VUR nephropathy is the first cause of pediatric hypertension and 10–20% of kids with VUR nephropathy will develop renal failure and need renal transplantation [19].

### Bulking agents and comparison

#### Teflon

Polytetrafluoroethylene (PTFE) or Teflon was the first material historically used for endoscopic treatment of VUR [20]. Long-term results (>20 years) of PTFE have been assessed [21–23]. Chertin et al. [22] reported absence of reflux in 95% of injected ureters on post-operative voiding cystourethrogram (VCUG) at a median follow-up of 13.5 years. Yucel et al. [23] demonstrated long-term durability and efficacy of PTFE, even in high-grade reflux (III-V), with a success rate of 68.4%. The main concern emerged as PTFE particles were found to have migrated in brain and lungs [24]. Teflon particles size ranges from 4 to 100 μm, with more than 90% smaller than 40 μm. Migration of particles as large as 80 μm was demonstrated in animal model [25]. Conversely, Miyakita and Puri reported no evidence of migration of PTFE particles to the brain in the following years [26, 27]. Despite the convincing long-term outcome, PTFE has been nearly abandoned.

#### Polydimethylsiloxane (Macroplastique)

This soft tissue bulking agent was based on elastomeric silicone incorporated into a patented device called Macroplastique (Congentix Medical, Orangeburg, NY, USA). It was highly viscous and no resorbable, requiring a specific administration device that can withstand high pressure [1]. These characteristics prevent shrinkage of the product and increase reliability [28]. Herz et al. [29] reported that correction by grade was 85, 84, 80, 45 and 0% for grades I to V, respectively. With repeat injection correction was 100, 92, 90 and 55% for grades I to IV, respectively. Most studies [30–32] reported no difference of efficacy compared to other substances but a prospective study [33] reported better success rate than Dx/Ha (90% vs 81%, *p* < 0.05) (Table 1). Most polydimethylsiloxane particles have diameters greater than 100 μm; but some are smaller than 80 μm, leading to possibility of long-distance migration [34]. Its use has been reduced since Deflux Food and Drug Administration (FDA) approval.

**Table 1:** Comparative studies reporting VUR resolution rate with different bulking agents

Study	VUR resolution rate			*P* value
	Polydimethylsiloxane	Dx/HA	Polyacrylate polyalcohol	
Kim et al. [25]	85%	82%	/	> 0.05
Bae et al. [26]	80.6%	78.6%	/	> 0.05
Oswald et al. [27]	86.2%	71.4%	/	= 0.05
Moore et al. [28]	90%	81%	/	< 0.05
Stredele et al. [38]	55%	81.5%	/	0.015
Karakus et al. [41]	/	70.3%	88.6%	0.007
Kocaoglu [42]	/	53.9%	80%	0.024
Garcia-Aparicio et al. [43]	/	77.3% (patients) 79.5% (ureters)	86.4% (patients) 85.3% (ureters)	0.698 0.557
Alizadeh et al. [44]	/	75.7%	92.2%	< 0.001
Bele et al. [45]	/	87.9%	94.7%	0.125
Warchol et al. [46]	/	63%	92.7%	n/a
Taskinlar et al. [47]	/	52.6% (single injection) 66.6% (multiple injection)	82% (single injection) 88.8% (multiple injection)	< 0.05 < 0.05

n/a = not available

#### Dx/HA (Deflux)

Deflux is a highly viscous gel of Dextranomere microspheres (80–250μm in diameter) in non-animal-stabilized hyaluronic acid, which acts as a carrier. In most cases, the implant volume ranges from 0.5 to 1.5 mL. It received FDA approval for UVR in 2002 and since then rapid increase in the use of EI , even as first-line treatment, was observed [35–37]. The overall success rate was between 70 and 90% (77% in a systematic review [38]) and vary according to VUR grade. However, long-term durability is debated. VCUG performed from 1 to 12 years postoperatively reported a recurrence rate from 12 to 54% [39–42].

Clinically and radiologically, Dx/HA exhibited the best results, giving better protection against UTIs and a better VUR cure rate compared to polydimethylsiloxane [43].

#### Polyacrylate polyalcohol copolymer (Vantris)

Polyacrylate Polyalcohol Copolymer (PPC) (Vantris, Promedon, Cordoba, Argentina), a non-biodegradable substance of synthetic origin, was introduced in 2010 [44]. The average diameter of particles is very high, average 320 nm, thus avoiding the risk of migration. The non-biodegradable nature allows formation of a fibrotic capsule, which provides better stability and long-term durability of the implant. High short-term efficacy (88.6–93.8% resolution rate) has been reported [44–46]. The VUR resolution rate of PPC resulted similar or superior to Dx/Ha after either single or multiple injection, as reported in some studies [46–52] (Table 1). PPC showed better results also when assessing persistent reflux after first injection (15 vs 33%) and after repeat injection (6 vs 18%) [48].

When assessing the clinical relevancy, post-injection febrile UTI rate between PPC (12%) and Dx/HA (14.6%) was not statistically significant (*p* = 0.54) [53]. The volume of bulking agent used for the injection was higher in case of Dx/HA rather than PPC [53]. A recent systematic review and meta-analysis indicated that PPC injection was associated with higher success rate, but concerningly, ureterovesical junction obstruction (UVJO) incidence was higher in the PPC group which might negate the possible benefits of PPC injection [54]. Additionally, the development of UVJO may also occur several months or even years after injection [55]. An animal study demonstrated that severe inflammation and fibrosis developed on injection site, probably due to continued foreign body reaction, presence of alcohol polymers, or larger particle sizes [56]. Subsequently, patients who undergo endoscopic treatment of PPC need long-term follow-up, despite reflux showing complete resolution [55].

### Techniques of injection and comparison

Subureteric injection (STING), described by Puri in 1984 [57], is the most adopted technique. The procedure consists in placing the needle 2–3 mm below the ureteric orifice at the 6 o’clock position, advancing it for 4–5 mm into the submucosal plane and creating a mound that elongates and coats the meatus. The overall success rate reported with this technique using Dx/HA was 69% at 12 months [58]. However, other centers have shown higher success rate, with resolution rate of 87.1% ureters after first injection, 11.3% after second and 1.6% after third injection [8]. The main limits of this technique were the relatively low success and possible caudal migration of the material.

A modification of the standard STING procedure, contributing to increased success rate, has been described as “ureteral repositioning and injection” (URI) by Capozza and Caione [59]. In the URI technique, the needle was inserted as for standard STING; subsequently the distal part of the ureter was raised and levered towards the lumen of the bladder; Dx/HA was then injected. This technique reported 91% VUR resolution rate and needed less material to inject compared to STING (0.4 vs 0.7 ml) [59].

The Hydrodistention Implantation Technique (HIT) consists of introducing the needle into the mucosa inside the ureteral tunnel. The main advantages of HIT over STING are the better visualization provided by hydrodistention, the more accurate placement of the needle and the better coaptation of the distal ureter and not only the orifice. HIT reported higher success rates than standard STING (max 92 vs 79%, *p* < 0.01) [60]. A recent meta-analysis [61] reported higher VUR resolution rate after HIT (82.5%) compared to STING (71.4%) [OR = 0.54, *p* < 0.0001]. A subgroup analysis showed that HIT had better outcomes than STING for both lower grade (I-III) [OR = 0.43; 95% CI 0.23–0.82; *P* = 0.01; *I*^2^ = 0%] and high-grade VUR (IV-V) [OR = 0.43; 95% CI 0.20–0.91; *P* = 0.03; *I*^2^ = 0%]. However, there was no statistical difference in the need of additional injections between STING and HIT groups.

Finally, double HIT is currently the most performed technique for endoscopic correction of VUR in the United States [62]. It consists of 2 intraluminal ureteric tunnel injections with hydrodistention. The 1^st^ injection of the bulking agent aims to coapt the detrusor tunnel whereas the 2^nd^ injection in more distal intramural tunnel leads to coaptation of the ureteric orifice. Double HIT requires higher injection volume, with a reported success rate of up to 93% [63, 64]. But it has potential drawbacks; in fact, multiple punctures of the mucosa may cause leakage of the injected material. Therefore, an additional distal ureter injection could be beneficial in the event of insufficient coaptation of the ureteric orifice.

### Predictive factors of success

#### VUR grade

A meta-analysis [38] revealed that pre-operative VUR grade was the single most important factor affecting the Dx/HA injection success rate. Success rate was 89% (grade I), 83% (grade II), 71% (grade III), 59% (grade IV) and 62% (grade V).

#### Technique of injection

HIT modification may increase the overall success rate (89 HIT vs 71% STING) [60]. Interestingly, the improvement in the results was stable over the time, even after the learning curve period, particularly in high grade VUR. Some studies reported no significant differences between the two techniques of injection [65].

#### Surgeon’s experience

A multivariate analysis confirmed that physician experience was an independent predictor of success rate after EI [66–68]. Three factors appear to be important predictors of reflux resolution after EI, that are surgeon’s experience, pre-operative VUR grade and the number of previous endoscopic treatment attempts [66]. A definite learning curve was seen as experience was gained with the technique [11]. The success rate increased after the first 20 cases and after the first 100 cases (60% to 80%). Then, the learning curve flattened after the initial 110 cases. This was seen especially for high-grade VUR and duplex system. The learning curve was shorter for low-grade VUR [11]. Factors of learning were the ability to clearly visualize the ureteral floor, choose the proper depth of injection, and select optimal pressure and volume of material required to create the mound.

#### Aspect of mound

A multivariate logistic regression analysis demonstrated that appearance of mound, correlated with outcomes [69]. The ability to create a satisfactory mound, that elevates and coapts the orifice, was the most important factor determining success of Dx/HA injection. Increasing reflux grade was associated with decreased likelihood of achieving a volcanic mound. Visualization of the mound around the ureterovesical junction on post-operative sonography could predict the success rate [70]. However, it is somewhat subjective. An online survey did not confirm the appearance of the mound and lack of hydrodistention at the completion of the procedure as reliable predictors of outcome [71].

#### Bowel bladder dysfunction (BBD)

The American Urological Association (AUA) 2010 Guidelines stated that the rate of cure following endoscopic therapy is less in children with (50%) than without BBD (89%) [72].

When controlling for pre-operative grade of VUR and BBD, the risk of persistent reflux was 2.8 times greater after subureteral injection of Dx/HA (95% CI 1.7–4.7, *p* < 0.0001) [73]. A long-term follow-up study confirmed that the only pre-operative condition affecting VUR recurrence was bladder dysfunction [74]. Endoscopic treatment with Dx/HA was reported to be similarly effective in patients with and without bladder dysfunction. Based on these data, BBD should not be considered a contraindication to endoscopic treatment [75] but should be treated before any surgical intervention for VUR is undertaken, especially voiding postponement, hyperactive bladder, dysfunctional voiding, and constipation. There are insufficient data to recommend a specific treatment regimen for BBD, but possible treatment options include behavioral therapy, biofeedback, anticholinergic medications, alpha blockers, and treatment of constipation [76].

#### Radiologic features

Ureteral diameter ratio (UDR) > 0.24, VUR during the early filling and delayed upper tract drainage at voiding are the most important predictive factors affecting the success of EI [12, 14].

### Specific situations

#### Paraureteral diverticulum

Paraureteral diverticulum (PUD) is usually an indication for surgical ureteral reimplantation because of the presumed underlying structural defect of the ureteral hiatus. However, EI has been done also in such cases, with an overall success rate of 68% after 1 implantation [77]. For injection in the lower PUD index, onset of reflux at late-filling or voiding phase on VCUG, higher pressure and volume on video urodynamics, and C position orifice were defined as positive predictive parameters for success [78]. Factors of success included size of diverticulum (< 2.6 times the ureteral diameter), late onset of reflux on VCUG, and position of the ureteral orifice. EI may be considered a treatment option in selected cases of PUD.

#### Ureteral duplication

A meta-analysis reported a lower overall success rate in ureteral duplication (50%) rather than in single systems (73%) regardless of VUR grade [79]. However, more recent studies reported better success rates (68.4–73%) after single injection, with the possibility of additional injections [7, 80, 81]. From the studies available, EI of bulking agents is highly successful in correcting mild-to-moderate VUR in duplex systems, with no reports of serious or clinically significant adverse effects. At a minimum, duplex systems would not seem to be a contraindication to the use of Deflux or any other bulking agent [81].

#### High grades

EI of Dx/HA is an efficient and safe long-term treatment for grade IV and V VUR and can easily be repeated in patients with treatment failure with high subsequent resolution rate. A recent study, including > 800 children, assessed long-term outcome (8 years) of 1287 EI using Deflux [6]. Resolution was reported in 70.4% of grade IV and 61.9% of grade V cases. Reflux resolved after a second injection in 20.1% and after a third injection in 10.4%. Failures after initial treatment were significantly more common in patients younger than 1 year and in individuals with renal scarring. No post-operative obstruction was observed, and no patient required ureteral reimplantation [6]. For some authors, EI may be the first line therapy whatever grade, but this may be decided on renal scars in high grades.

### Controversies

#### EI is currently the first-line therapy for VUR

From 2002 to 2006, dramatic increase of utilization of EI was registered in the US [36, 37]. The mean number of injections per institution yearly increased from 17 to 66 from 2002 to 2004 or 288% [37]. After 2011, there was a trend toward decreasing intervention for primary VUR, which appeared to be due to decreased use of injection therapy [82]. This change was attributable to top-down approach, with less low grade VUR detected, more conservative treatment of low-grade VUR and unchanged number of ureteral reimplantation for high-grade VUR.

EI is currently the first-line therapy for children with grade III–V primary reflux in many institutions worldwide. In contrast to continuous antibiotic prophylaxis, this procedure offers immediate cure with resolution rates ranging from 77 to 83% and is independent of patient or parent compliance. It is an efficient and safe long-term option also for grade IV and V VUR with success rates of 70% after the first injection, which can be easily repeated in cases of failure with a high subsequent resolution rate [8, 83].

#### Long-term durability

Decreased success rate was reported at long-term follow-up. Even in patients with immediate resolution of VUR, 26% recurrence of VUR was reported after 1 year [42]. Although the reflux resolution rates at initial post-operative VCUG approach those of open surgery, the significant late failure rate at 1 year warrants long-term follow-up. The Swedish reflux trial [84] showed 20% recurrence rate after 2 years with VUR grade > III. This might be probably explained by migration of material accelerated by BBD.

#### Ureteral obstruction

Early and delayed ureteral obstructions have been reported following EI, although the incidence was still lower than with open surgery [85]. Most cases resolved after temporary double-J stenting, but some required open reimplantation because of inflammatory foreign body reaction. Formation of a pseudocapsule and calcification are known histologic changes at the injection site, and are more frequent than expected (9%), especially in children younger than 3 years [86–88]. It may be misdiagnosed with lithiasis and lead to unnecessary ureteroscopy [89]. Ureteral obstruction remains a rare complication after endoscopic correction of VUR, generally reported in less than 1% of treated cases, which appears to be independent of the injected substance, volume, and technique [90]. However, long-term follow-up (5 years) is recommended as asymptomatic or delayed obstruction can occur, potentially leading to loss of renal function [91].

#### Real efficacy of EI

- *In the occurrence of UTI*: Elder et al. [92] reported reduced number of UTIs per year after EI with Dx/HA vs antibiotic prophylaxis (0.08 vs 0.28), supporting a role for Dx/HA as first-line treatment option for patients with VUR. But Swedish trial [93] demonstrated that the rate of febrile UTIs was lower with EI (23%) compared to surveillance (57%) but did not differ between EI (23%) and antibiotic prophylaxis (19%). Additionally, EI reported no reduction of UTIs in boys older than 1 year with dilating VUR.
- *In preventing renal damage*: totally unknown, both on renal deterioration and scarring. Deterioration of renal function still occurs in 9% of patients following EI [94]. The Swedish trial [95] showed that antimicrobial therapy had the lowest incidence of renal scarring after 2 years and the rate of new renal damage was not different between EI, antibiotic prophylaxis and surveillance. The incidence of new renal damage was low in boys but significantly higher in girls. There was also significant correlation between recurrent febrile UTIs and appearance of new renal damage in girls [95].

## Discussion

In the last years, there was a paradigm shift in the treatment of VUR. Currently, the treatment focus is no longer the presence or not of reflux. The goal of management is preservation of renal function. Nowadays, VUR is considered only a radiological sign and is treated because it is a risk factor for febrile UTIs (fUTIs). Recurrent fUTIs can cause an acquired damage (renal scars) that might add up to a congenital damage (renal dysplasia), if present. In terms of treatment, we have a wide range of options, that go from don’t make diagnosis to observation with or without CAP to surgical treatment with either EI or ureteral reimplantation, which can be performed using either open approach or minimally invasive surgery. The idea that “don’t make diagnosis” could be an option was the base to develop the so called “top-down approach”, according to which, after the first fUTI, if there are no signs of parenchymal involvement of the infection, we don’t have to go further with VCUG to check the presence of VUR. The European Association of Urology (EAU) developed guidelines on VUR in children [96], in which the variables of relevance for the management of VUR are symptoms (fUTIs); gender; toilet training status; presence of BBD; VUR grade (high vs low) and status of kidney parenchyma (normal vs abnormal). To these variables, we would suggest adding parental preference, that plays a key role in the decision-making strategy.

But when endoscopic treatment should be proposed? Most authors offer this treatment option to patients with breakthrough fUTIs, or fUTIs after discontinuation of CAP, or first fUTI in toilet trained patients, or poor parental compliance to CAP. The last 2019 Cochrane Review [97] reported that despite significant reduction in repeat episodes of fUTIs reported by surgery, there were no differences between surgery and long-term low-dose antibiotic use in either symptomatic UTI or renal damage. Correcting VUR using endoscopic approaches would theoretically reduce the risks of adverse events associated with surgery.

One of the most debated aspects of endoscopic treatment is the material to be used. The ideal material should be malleable to make the injection easier; should be stable after injection to ensure the durability of the implant; should be biocompatible to avoid the risk of obstruction secondary to any local inflammatory reaction; and should have no risks of distant migration in the body. To date, no ideal material is still available. Many materials have been proposed, utilized, and then discouraged along the last 30 years. Currently, the 2 most used materials for injection are Deflux and Vantris. The first is absorbable, easier to inject, has lower risk of obstruction, but can lose efficacy over time. The second is non-absorbable, more difficult to inject, has higher risk of obstruction, but it is potentially more durable.

Regarding the technique of injection, there are 2 major procedures: one is the sub-ureteral injection (STING) described by Puri and the second is the intra-ureteral injection (HIT) after hydrodistention of the orifice described by Kirsch. In the original paper by Kirsch [60], the use of HIT reported higher success rate (89%) than STING (71%) and this was more evident in high-grade reflux (grade III and IV). These results were not duplicated in the following studies. In a multivariate analysis [68], there was a trend toward improved results with ureteral hydrodistention combined with intra-ureteral injection, although this did not achieve statistical significance. Only reflux grade and surgeon’s experience were independently predictive of injection success in patients with primary, uncomplicated VUR.

Beside STING and HIT, several other techniques have been described in the literature. Most of them can be combined; multiple intra- and sub-ureteral injections can be performed to obtain a mountain range effect. Some of these techniques can be useful in specific circumstances such as VUR in paraureteral diverticulum, ureterocele, renal transplantation or after ureteral reimplant.

In any case, the surgeon’s experience is the key for the success [66–68]. Other key factors to success are use of adequate material and instrumentation and selection of appropriate technique, depending on the ureteral orifice. If the ureteral orifice has “golf hole” appearance, intra-ureteral injection should be more suited; in case of “horseshoe” appearance of the ureteral hiatus, URI technique could be more appropriate to reconstruct a true flap-valve mechanism, without the risk of ureteral obstruction [59].

Obviously, endoscopic treatment may also have complications. The most common is ureteral obstruction. It was reported in < 1% after Deflux injection, but it is possibly higher after treatment with Vantris [54]. Based upon this evidence, less material should be implanted if Vantris is used. Obstruction seems to be more common in cases with dysfunctional bladder and tortuous dysplastic ureter.

Analyzing the available literature, few studies of low methodological quality have investigated if endoscopic correction may make significant difference to number of symptomatic or fUTIs or in new or progressive renal damage. So, future research should give definitive answers.

## Conclusion

EI represents a valid treatment option for pediatric VUR; it is easy, reproducible, with short learning curve and low-morbidity profile. It reported satisfactory outcomes with resolution rates ranging from 69 to 100%. Obviously, the success rate may be influenced by several factors. Recently, it is adopted as first-line therapy also in high-grade reflux or complex anatomy such as duplex, bladder diverticula, ectopic ureters. The ideal material and technique of injection has not yet clearly established, but the choice is still dependent on surgeon’s preference and experience.

## Data Availability

The data that support the findings of this study are available from the corresponding author, Maria Escolino, upon reasonable request.
